# Hydroxonium creatininium bis­(pyridine-2,6-dicarboxyl­ato-κ^3^ *O* ^2^,*N*,*O* ^6^)cobaltate(II) trihydrate

**DOI:** 10.1107/S1600536809021837

**Published:** 2009-06-27

**Authors:** Hossein Aghabozorg, Zohreh Derikvand, Jafar Attar Gharamaleki, Mohammad Yousefi

**Affiliations:** a Faculty of Chemistry, Islamic Azad University, North Tehran Branch, Tehran, Iran; b Department of Chemistry, Faculty of Science, Islamic Azad University, Khorramabad Branch, Khorramabad, Iran; c Young Researchers Club, Islamic Azad University, North Tehran Branch, Tehran, Iran; d Department of Chemistry, Islamic Azad University, Shahr-e-Rey Branch, Tehran, Iran

## Abstract

The title compound, (C_4_H_8_N_3_O)(H_3_O)[Co(C_7_H_3_NO_4_)_2_]·3H_2_O, contains a protonated creatininium cation, a hydrox­onium (H_3_O)^+^ cation, a [Co(pydc)_2_]^2−^ (pydcH_2_ = pyridine-2,6-dicarboxylic acid) complex anion, and three uncoordinated water mol­ecules. The Co^II^ atom is coordinated by four O and two N atoms from two pydc ligands in a distorted octa­hedral environment. The structure also contains three uncoordinated water mol­ecules. Extensive inter­molecular O—H⋯O, N—H⋯O and C—H⋯O hydrogen bonds, π–π stacking inter­actions [centroid–centroid distances = 3.565 (14) and 3.425 (14) Å] and O⋯π inter­actions [O⋯centroid distance = 3.480 (2) Å] connect the various components in the crystal structure.

## Related literature

For related structures, see: Aghabozorg, Derikvand *et al.* (2008 ▶); Aghabozorg, Ramezanipour *et al.* (2008 ▶); Moghimi *et al.* (2004 ▶, 2005 ▶). For a review article on proton-transfer agents and their metal complexes, see: Aghabozorg, Manteghi *et al.* (2008 ▶). For the isotypic Ni compound, see: Attar Gharamaleki *et al.* (2009 ▶).
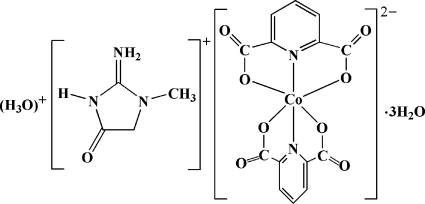


## Experimental

### 

#### Crystal data

(C_4_H_8_N_3_O)(H_3_O)[Co(C_7_H_3_NO_4_)_2_]·3H_2_O
*M* *_r_* = 576.34
Triclinic, 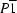

*a* = 8.0937 (10) Å
*b* = 10.7389 (13) Å
*c* = 13.5976 (17) Å
α = 104.811 (2)°
β = 90.267 (2)°
γ = 92.415 (1)°
*V* = 1141.4 (2) Å^3^
*Z* = 2
Mo *K*α radiation
μ = 0.83 mm^−1^
*T* = 120 K
0.18 × 0.12 × 0.09 mm

#### Data collection

Bruker SMART 1000 CCD diffractometer
Absorption correction: multi-scan (*SADABS*; Sheldrick, 1996 ▶) *T*_min_ = 0.889, *T*_max_ = 0.930
11652 measured reflections
5488 independent reflections
4149 reflections with *I* > 2σ(*I*)
*R*_int_ = 0.025

#### Refinement

*R*[*F* ^2^ > 2σ(*F* ^2^)] = 0.045
*wR*(*F* ^2^) = 0.097
*S* = 1.02
5488 reflections
335 parameters
H-atom parameters constrained
Δρ_max_ = 0.77 e Å^−3^
Δρ_min_ = −0.45 e Å^−3^

### 

Data collection: *SMART* (Bruker, 2007 ▶); cell refinement: *SAINT-Plus* (Bruker, 2007 ▶); data reduction: *SAINT-Plus*; program(s) used to solve structure: *SHELXTL* (Sheldrick, 2008 ▶); program(s) used to refine structure: *SHELXTL*; molecular graphics: *SHELXTL* and *Mercury* (Macrae *et al.*, 2006 ▶); software used to prepare material for publication: *SHELXTL*.

## Supplementary Material

Crystal structure: contains datablocks I, global. DOI: 10.1107/S1600536809021837/hy2201sup1.cif

Structure factors: contains datablocks I. DOI: 10.1107/S1600536809021837/hy2201Isup2.hkl

Additional supplementary materials:  crystallographic information; 3D view; checkCIF report

## Figures and Tables

**Table 1:** Selected bond lengths (Å)

Co1—N1	2.029 (2)
Co1—N2	2.031 (2)
Co1—O8	2.1273 (18)
Co1—O4	2.1389 (18)
Co1—O5	2.1904 (18)
Co1—O1	2.2239 (19)

**Table 2:** Hydrogen-bond geometry (Å, °)

*D*—H⋯*A*	*D*—H	H⋯*A*	*D*⋯*A*	*D*—H⋯*A*
N3—H3*A*⋯O1*W*^i^	0.88	1.86	2.716 (3)	164
N5—H5*A*⋯O5	0.88	2.15	2.882 (3)	141
N5—H5*B*⋯O3^ii^	0.88	1.96	2.764 (3)	152
O1*W*—H1⋯O4^iii^	0.85	1.95	2.782 (3)	166
O1*W*—H2⋯O3*W*^iv^	0.85	1.85	2.673 (3)	164
O2*W*—H3⋯O4*W*^v^	0.85	1.70	2.522 (3)	163
O2*W*—H4⋯O6^vi^	0.85	1.64	2.481 (3)	170
O2*W*—H5⋯O2	0.85	1.71	2.537 (3)	164
O3*W*—H6⋯O7^iii^	0.85	1.93	2.778 (3)	172
O3*W*—H7⋯O9^vii^	0.85	2.22	2.948 (3)	144
O4*W*—H8⋯O7^viii^	0.85	1.84	2.680 (3)	169
O4*W*—H9⋯O1	0.85	1.87	2.718 (3)	172
C3—H3*B*⋯O9^vii^	0.95	2.37	3.301 (3)	165
C4—H4*A*⋯O8^ix^	0.95	2.43	3.252 (3)	145
C18—H18*C*⋯O7^iii^	0.98	2.60	3.535 (4)	160

Symmetry codes: (i) 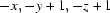; (ii) 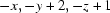; (iii) 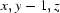; (iv) 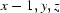; (v) 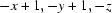; (vi) 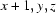; (vii) 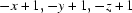; (viii) 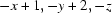; (ix) 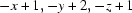.

**Fig. 1.**
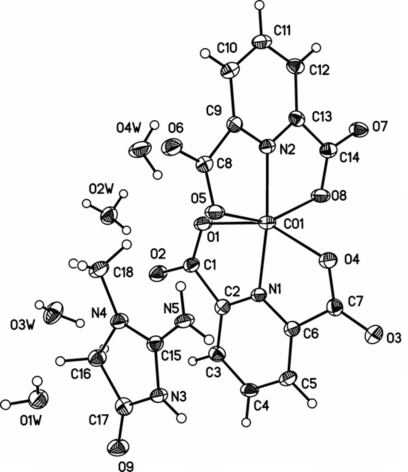
Molecular structure of the title compound. Displacement ellipsoids are drawn at the 50% probability level.

**Fig. 2.**
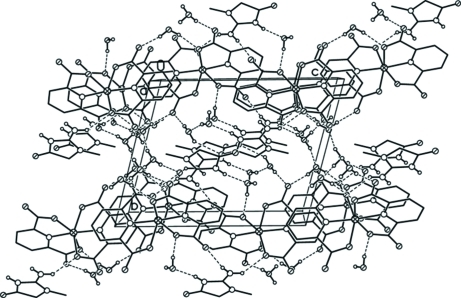
Packing diagram of the title compound. Hydrogen bonds are shown by dashed lines.

**Fig. 3.**
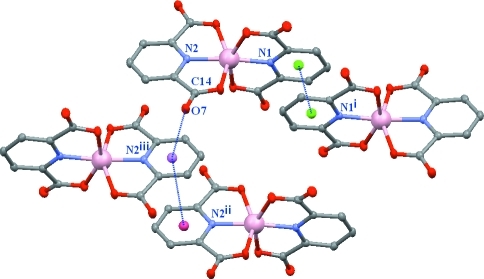
The π–π and O···π stacking interactions in the title compound. The centroid–centroid distances are 3.425 (14) Å between Cg1 (N1, C2–C6) and Cg1i, and 3.565 (14) Å between Cg2ii (N2ii, C9ii–C13ii) and Cg2iii. The O7···Cg2iii distance is 3.480 (2) Å. [Symmetry codes: (i) 1-x, 2-y, 1-z; (ii) 1+x, y, z; (iii) 1-x, 2-y, -z.]

## supplementary crystallographic information

### Comment

We have previously reported some compounds containing creatinine (creat), pyridine-2,6-dicarboxylic acid (pydcH_2_) and various metals, such as (creatH)(pydcH).H_2_O (Moghimi *et al.*, 2004), (creatH)_2_[Bi(pydc)_2_]_2_.4H_2_O (Moghimi *et al.*, 2005), (creatH)[Zn(pydc)(pydcH)].4H_2_O (Aghabozorg, Ramezanipour *et al.*, 2008) and (creatH)[Cr(pydc)_2_](pydcH_2_).6H_2_O (Aghabozorg, Derikvand *et al.,* 2008). For more details and related literature see our recent review article (Aghabozorg, Manteghi *et al.*, 2008).

We describe here the crystal structure of the title compound. The compound contains a [Co(pydc)_2_]^2-^ anion, a (creatH)^+^ and a (H_3_O)^+^ cation, and three uncoordinated water molecules (Fig. 1). In the anion, the Co^II^ atom is six-coordinated by two N atoms (N1 and N2) and four O atoms (O1, O4, O5 and O8) from the carboxylate groups of two (pydc)^2-^ ligands, with the bond length range of 2.029 (2)–2.2239 (19) Å (Table 1). The N1—Co1—N2 [171.90 (8)°], O8—Co1—O5 [151.26 (7)°] and O4—Co1—O1 [152.09 (7)°] angles show that the four carboxylate groups of the two (pydc)^2-^ ligands orient in a flattened tetrahedral arrangement around the central atom. The coordination environment around Co^II^ is distorted octahedral. The O8—Co1—O4—C7 and O1—Co1—O8—C14 torsion angles are -95.15 (17)° and 95.48 (19)°, respectively, thus it can be concluded that two (pydc)^2-^ ligands are almost perpendicular to each other. The intermolecular forces in the structure could be divided in three main branches, ionic interactions which gather principal anionic complex and counter cation together, *X*—H···O hydrogen bonds (Fig. 2 and Table 2), where *X*= O, N, C, and O···π, π–π stacking interactions. The π–π stacking interactions between the pyridyl rings, with centroid–centroid distances of 3.565 (14) and 3.425 (14) Å, and the O···π interaction between the carboxylate O atom and pyridyl ring, with an O···centroid distance of 3.480 (2)Å are observed (Fig. 3). Ion pairing, π–π stacking interactions and extensive intermolecular hydrogen bonds connected the various components into a supramolecular structure.

### Experimental

The reaction between pyridine-2,6-dicarboxylic acid (100 mg, 1 mmol) in 10 ml water, cratinine (110 mg, 1 mmol) in 20 ml water and Co(NO_3_)_2_.6H_2_O (87 mg, 0.5 mmol) in 5 ml water at a 2:2:1 molar ratio gave a red compound after slow evaporation of the solvent at the room temperature. The crystals obtained were stable in air.

### Refinement

H atoms on O and N atoms were found from difference Fourier maps. H atoms on C atoms were positioned geometrically. All H atoms were refined in riding models, with *U*_iso_(H) = 1.2(1.5 for methyl)*U*_eq_(C,N) or 1.5*U*_eq_(O).

### Crystal data

**Table:**

(C_4_H_8_N_3_O)(H_3_O)[Co(C_7_H_3_NO_4_)_2_]·3H_2_O	*Z* = 2
*M**_r_* = 576.34	*F*(000) = 594
Triclinic, *P*1	*D*_x_ = 1.677 Mg m^−^^3^
Hall symbol: -P 1	Mo *K*α radiation, λ = 0.71073 Å
*a* = 8.0937 (10) Å	Cell parameters from 843 reflections
*b* = 10.7389 (13) Å	θ = 3–27°
*c* = 13.5976 (17) Å	µ = 0.83 mm^−^^1^
α = 104.811 (2)°	*T* = 120 K
β = 90.267 (2)°	Prism, red
γ = 92.415 (1)°	0.18 × 0.12 × 0.09 mm
*V* = 1141.4 (2) Å^3^	

### Data collection

**Table:**

Bruker SMART 1000 CCD diffractometer	5488 independent reflections
Radiation source: fine-focus sealed tube	4149 reflections with *I* > 2σ(*I*)
graphite	*R*_int_ = 0.025
φ and ω scans	θ_max_ = 28.0°, θ_min_ = 1.6°
Absorption correction: multi-scan (*SADABS*; Sheldrick, 1996)	*h* = −10→10
*T*_min_ = 0.889, *T*_max_ = 0.930	*k* = −14→14
11652 measured reflections	*l* = −17→17

### Refinement

**Table:**

Refinement on *F*^2^	Primary atom site location: structure-invariant direct methods
Least-squares matrix: full	Secondary atom site location: difference Fourier map
*R*[*F*^2^ > 2σ(*F*^2^)] = 0.045	Hydrogen site location: mixed
*wR*(*F*^2^) = 0.097	H-atom parameters constrained
*S* = 1.02	*w* = 1/[σ^2^(*F*_o_^2^) + (0.015*P*)^2^ + 2.6*P*] where *P* = (*F*_o_^2^ + 2*F*_c_^2^)/3
5488 reflections	(Δ/σ)_max_ < 0.001
335 parameters	Δρ_max_ = 0.77 e Å^−^^3^
0 restraints	Δρ_min_ = −0.45 e Å^−^^3^

### Fractional atomic coordinates and isotropic or equivalent isotropic displacement parameters (Å^2^)

**Table:**

	*x*	*y*	*z*	*U*_iso_*/*U*_eq_	
Co1	0.26506 (4)	0.93000 (3)	0.23131 (3)	0.01942 (10)	
O1	0.4562 (2)	0.78341 (18)	0.19033 (13)	0.0230 (4)	
O2	0.6104 (2)	0.65616 (18)	0.25814 (14)	0.0252 (4)	
O3	0.0940 (2)	1.13617 (18)	0.50661 (14)	0.0254 (4)	
O4	0.1083 (2)	1.04931 (18)	0.33852 (13)	0.0232 (4)	
O5	0.0722 (2)	0.78287 (17)	0.16490 (13)	0.0220 (4)	
O6	−0.0601 (2)	0.69039 (18)	0.01715 (13)	0.0249 (4)	
O7	0.4810 (2)	1.23340 (18)	0.13445 (13)	0.0238 (4)	
O8	0.4131 (2)	1.09779 (18)	0.22942 (13)	0.0241 (4)	
O9	0.2979 (2)	0.47606 (19)	0.53766 (14)	0.0287 (4)	
N1	0.3283 (2)	0.89101 (19)	0.36453 (15)	0.0161 (4)	
N2	0.2268 (2)	0.9517 (2)	0.08903 (15)	0.0170 (4)	
N3	0.1336 (3)	0.5901 (2)	0.45644 (15)	0.0181 (4)	
H3A	0.0801	0.6346	0.5091	0.022*	
N4	0.2130 (3)	0.5206 (2)	0.29648 (16)	0.0202 (4)	
N5	0.0027 (3)	0.6690 (2)	0.33142 (15)	0.0210 (5)	
H5A	−0.0074	0.6703	0.2672	0.025*	
H5B	−0.0602	0.7165	0.3776	0.025*	
C1	0.5092 (3)	0.7418 (2)	0.26371 (18)	0.0188 (5)	
C2	0.4403 (3)	0.8035 (2)	0.36658 (19)	0.0180 (5)	
C3	0.4860 (3)	0.7762 (2)	0.45730 (19)	0.0198 (5)	
H3B	0.5660	0.7143	0.4584	0.024*	
C4	0.4112 (3)	0.8422 (2)	0.54679 (19)	0.0203 (5)	
H4A	0.4382	0.8245	0.6098	0.024*	
C5	0.2971 (3)	0.9341 (2)	0.54291 (19)	0.0206 (5)	
H5C	0.2460	0.9807	0.6031	0.025*	
C6	0.2589 (3)	0.9566 (2)	0.44902 (18)	0.0174 (5)	
C7	0.1427 (3)	1.0555 (2)	0.43192 (19)	0.0190 (5)	
C8	0.0403 (3)	0.7707 (2)	0.07148 (18)	0.0191 (5)	
C9	0.1295 (3)	0.8660 (2)	0.02324 (18)	0.0177 (5)	
C10	0.1127 (3)	0.8709 (3)	−0.07723 (19)	0.0201 (5)	
H10A	0.0447	0.8087	−0.1240	0.024*	
C11	0.1983 (3)	0.9690 (3)	−0.10736 (19)	0.0222 (5)	
H11A	0.1896	0.9743	−0.1759	0.027*	
C12	0.2966 (3)	1.0597 (3)	−0.03808 (19)	0.0200 (5)	
H12A	0.3538	1.1283	−0.0578	0.024*	
C13	0.3090 (3)	1.0472 (2)	0.06107 (19)	0.0180 (5)	
C14	0.4103 (3)	1.1339 (2)	0.14799 (19)	0.0199 (5)	
C15	0.1105 (3)	0.5968 (2)	0.35806 (18)	0.0182 (5)	
C16	0.3147 (3)	0.4547 (3)	0.35453 (19)	0.0221 (5)	
H16A	0.4336	0.4783	0.3505	0.027*	
H16B	0.2976	0.3599	0.3303	0.027*	
C17	0.2520 (3)	0.5040 (3)	0.4611 (2)	0.0213 (5)	
C18	0.2037 (4)	0.4869 (3)	0.18563 (19)	0.0279 (6)	
H18A	0.2195	0.5652	0.1617	0.042*	
H18B	0.0951	0.4457	0.1627	0.042*	
H18C	0.2903	0.4272	0.1580	0.042*	
O1W	−0.0036 (2)	0.29618 (18)	0.36045 (14)	0.0249 (4)	
H1	0.0162	0.2166	0.3476	0.037*	
H2	−0.0856	0.3008	0.3225	0.037*	
O2W	0.7786 (2)	0.55028 (19)	0.10484 (14)	0.0293 (4)	
H3	0.7269	0.4912	0.0603	0.044*	
H4	0.8232	0.6010	0.0728	0.044*	
H5	0.7115	0.5907	0.1481	0.044*	
O3W	0.7461 (3)	0.3583 (2)	0.25576 (16)	0.0362 (5)	
H6	0.6588	0.3236	0.2235	0.054*	
H7	0.7240	0.4284	0.2983	0.054*	
O4W	0.3941 (3)	0.6406 (2)	−0.00313 (15)	0.0468 (7)	
H8	0.4336	0.6891	−0.0386	0.070*	
H9	0.4122	0.6782	0.0592	0.070*	

### Atomic displacement parameters (Å^2^)

**Table:**

	*U*^11^	*U*^22^	*U*^33^	*U*^12^	*U*^13^	*U*^23^
Co1	0.02341 (19)	0.02192 (19)	0.01396 (17)	0.00133 (14)	−0.00051 (13)	0.00646 (13)
O1	0.0280 (10)	0.0275 (10)	0.0147 (9)	0.0058 (8)	0.0015 (7)	0.0067 (7)
O2	0.0268 (10)	0.0296 (10)	0.0195 (9)	0.0106 (8)	0.0029 (8)	0.0051 (8)
O3	0.0299 (10)	0.0242 (10)	0.0208 (9)	0.0052 (8)	0.0040 (8)	0.0027 (8)
O4	0.0289 (10)	0.0246 (10)	0.0173 (9)	0.0063 (8)	0.0022 (7)	0.0065 (7)
O5	0.0269 (10)	0.0249 (10)	0.0152 (9)	−0.0037 (8)	−0.0011 (7)	0.0075 (7)
O6	0.0303 (10)	0.0264 (10)	0.0168 (9)	−0.0080 (8)	−0.0021 (8)	0.0048 (8)
O7	0.0279 (10)	0.0247 (10)	0.0197 (9)	−0.0077 (8)	−0.0030 (8)	0.0091 (8)
O8	0.0328 (10)	0.0253 (10)	0.0155 (9)	−0.0052 (8)	−0.0017 (8)	0.0086 (7)
O9	0.0327 (11)	0.0354 (11)	0.0201 (9)	0.0093 (9)	−0.0025 (8)	0.0100 (8)
N1	0.0173 (10)	0.0173 (10)	0.0136 (9)	−0.0022 (8)	0.0000 (8)	0.0042 (8)
N2	0.0173 (10)	0.0195 (11)	0.0142 (10)	0.0018 (8)	−0.0002 (8)	0.0043 (8)
N3	0.0199 (10)	0.0216 (11)	0.0133 (10)	0.0024 (8)	0.0011 (8)	0.0050 (8)
N4	0.0224 (11)	0.0230 (11)	0.0153 (10)	0.0054 (9)	−0.0002 (8)	0.0047 (9)
N5	0.0255 (11)	0.0262 (12)	0.0116 (10)	0.0025 (9)	0.0010 (8)	0.0051 (9)
C1	0.0199 (12)	0.0205 (13)	0.0150 (11)	−0.0018 (10)	−0.0008 (9)	0.0033 (10)
C2	0.0167 (12)	0.0184 (12)	0.0179 (12)	−0.0009 (9)	0.0010 (9)	0.0035 (10)
C3	0.0193 (12)	0.0214 (13)	0.0193 (12)	−0.0009 (10)	−0.0028 (10)	0.0067 (10)
C4	0.0242 (13)	0.0231 (13)	0.0141 (11)	−0.0049 (10)	−0.0038 (10)	0.0067 (10)
C5	0.0227 (13)	0.0231 (13)	0.0141 (12)	−0.0035 (10)	0.0020 (10)	0.0021 (10)
C6	0.0172 (12)	0.0178 (12)	0.0165 (12)	−0.0023 (9)	0.0003 (9)	0.0037 (10)
C7	0.0180 (12)	0.0178 (12)	0.0219 (12)	0.0009 (10)	0.0031 (10)	0.0062 (10)
C8	0.0210 (12)	0.0194 (12)	0.0166 (12)	0.0011 (10)	0.0013 (10)	0.0039 (10)
C9	0.0185 (12)	0.0175 (12)	0.0171 (12)	0.0023 (10)	0.0020 (9)	0.0042 (10)
C10	0.0187 (12)	0.0246 (13)	0.0155 (12)	0.0009 (10)	−0.0006 (9)	0.0028 (10)
C11	0.0250 (13)	0.0286 (14)	0.0143 (12)	0.0030 (11)	0.0002 (10)	0.0074 (10)
C12	0.0188 (12)	0.0238 (13)	0.0193 (12)	0.0036 (10)	0.0032 (10)	0.0087 (10)
C13	0.0160 (12)	0.0194 (12)	0.0201 (12)	0.0037 (9)	0.0018 (9)	0.0074 (10)
C14	0.0180 (12)	0.0239 (13)	0.0181 (12)	0.0014 (10)	0.0003 (10)	0.0059 (10)
C15	0.0194 (12)	0.0185 (12)	0.0158 (12)	−0.0035 (10)	0.0001 (9)	0.0031 (10)
C16	0.0225 (13)	0.0246 (13)	0.0195 (12)	0.0042 (11)	−0.0009 (10)	0.0056 (10)
C17	0.0197 (12)	0.0216 (13)	0.0227 (13)	0.0003 (10)	−0.0019 (10)	0.0062 (10)
C18	0.0381 (16)	0.0293 (15)	0.0149 (12)	0.0067 (12)	0.0010 (11)	0.0024 (11)
O1W	0.0284 (10)	0.0249 (10)	0.0216 (9)	0.0065 (8)	−0.0015 (8)	0.0052 (8)
O2W	0.0354 (11)	0.0256 (10)	0.0246 (10)	−0.0025 (9)	0.0084 (8)	0.0027 (8)
O3W	0.0351 (12)	0.0370 (12)	0.0304 (11)	0.0082 (10)	−0.0113 (9)	−0.0037 (9)
O4W	0.0845 (19)	0.0357 (13)	0.0170 (10)	−0.0282 (12)	0.0008 (11)	0.0061 (9)

### Geometric parameters (Å, °)

**Table:**

Co1—N1	2.029 (2)	C3—H3B	0.9500
Co1—N2	2.031 (2)	C4—C5	1.390 (4)
Co1—O8	2.1273 (18)	C4—H4A	0.9500
Co1—O4	2.1389 (18)	C5—C6	1.394 (3)
Co1—O5	2.1904 (18)	C5—H5C	0.9500
Co1—O1	2.2239 (19)	C6—C7	1.509 (3)
O1—C1	1.273 (3)	C8—C9	1.511 (3)
O2—C1	1.245 (3)	C9—C10	1.387 (3)
O3—C7	1.233 (3)	C10—C11	1.386 (4)
O4—C7	1.283 (3)	C10—H10A	0.9500
O5—C8	1.268 (3)	C11—C12	1.388 (4)
O6—C8	1.249 (3)	C11—H11A	0.9500
O7—C14	1.247 (3)	C12—C13	1.392 (3)
O8—C14	1.263 (3)	C12—H12A	0.9500
O9—C17	1.215 (3)	C13—C14	1.516 (3)
N1—C6	1.325 (3)	C16—C17	1.509 (4)
N1—C2	1.338 (3)	C16—H16A	0.9900
N2—C9	1.334 (3)	C16—H16B	0.9900
N2—C13	1.335 (3)	C18—H18A	0.9800
N3—C15	1.370 (3)	C18—H18B	0.9800
N3—C17	1.371 (3)	C18—H18C	0.9800
N3—H3A	0.8800	O1W—H1	0.8500
N4—C15	1.331 (3)	O1W—H2	0.8500
N4—C18	1.458 (3)	O2W—H3	0.8500
N4—C16	1.459 (3)	O2W—H4	0.8500
N5—C15	1.301 (3)	O2W—H5	0.8500
N5—H5A	0.8800	O3W—H6	0.8500
N5—H5B	0.8800	O3W—H7	0.8500
C1—C2	1.507 (3)	O4W—H8	0.8500
C2—C3	1.391 (3)	O4W—H9	0.8500
C3—C4	1.397 (4)		
			
N1—Co1—N2	171.90 (8)	N1—C6—C7	113.5 (2)
N1—Co1—O8	104.11 (7)	C5—C6—C7	125.5 (2)
N2—Co1—O8	76.44 (7)	O3—C7—O4	126.1 (2)
N1—Co1—O4	77.02 (8)	O3—C7—C6	118.5 (2)
N2—Co1—O4	111.08 (8)	O4—C7—C6	115.4 (2)
O8—Co1—O4	88.59 (7)	O6—C8—O5	126.3 (2)
N1—Co1—O5	104.55 (7)	O6—C8—C9	117.6 (2)
N2—Co1—O5	75.49 (7)	O5—C8—C9	116.1 (2)
O8—Co1—O5	151.26 (7)	N2—C9—C10	121.4 (2)
O4—Co1—O5	95.77 (7)	N2—C9—C8	112.8 (2)
N1—Co1—O1	75.10 (7)	C10—C9—C8	125.8 (2)
N2—Co1—O1	96.82 (7)	C11—C10—C9	118.0 (2)
O8—Co1—O1	99.21 (7)	C11—C10—H10A	121.0
O4—Co1—O1	152.09 (7)	C9—C10—H10A	121.0
O5—Co1—O1	90.12 (7)	C10—C11—C12	120.4 (2)
C1—O1—Co1	115.11 (16)	C10—C11—H11A	119.8
C7—O4—Co1	114.49 (16)	C12—C11—H11A	119.8
C8—O5—Co1	115.09 (16)	C11—C12—C13	118.1 (2)
C14—O8—Co1	116.51 (16)	C11—C12—H12A	121.0
C6—N1—C2	121.3 (2)	C13—C12—H12A	121.0
C6—N1—Co1	118.26 (17)	N2—C13—C12	120.9 (2)
C2—N1—Co1	120.43 (16)	N2—C13—C14	112.3 (2)
C9—N2—C13	121.1 (2)	C12—C13—C14	126.8 (2)
C9—N2—Co1	119.81 (16)	O7—C14—O8	125.7 (2)
C13—N2—Co1	118.89 (16)	O7—C14—C13	118.6 (2)
C15—N3—C17	110.3 (2)	O8—C14—C13	115.6 (2)
C15—N3—H3A	124.8	N5—C15—N4	126.3 (2)
C17—N3—H3A	124.8	N5—C15—N3	123.3 (2)
C15—N4—C18	125.2 (2)	N4—C15—N3	110.4 (2)
C15—N4—C16	110.1 (2)	N4—C16—C17	102.3 (2)
C18—N4—C16	123.7 (2)	N4—C16—H16A	111.3
C15—N5—H5A	120.0	C17—C16—H16A	111.3
C15—N5—H5B	120.0	N4—C16—H16B	111.3
H5A—N5—H5B	120.0	C17—C16—H16B	111.3
O2—C1—O1	126.4 (2)	H16A—C16—H16B	109.2
O2—C1—C2	118.0 (2)	O9—C17—N3	125.7 (2)
O1—C1—C2	115.6 (2)	O9—C17—C16	127.5 (2)
N1—C2—C3	121.2 (2)	N3—C17—C16	106.8 (2)
N1—C2—C1	113.7 (2)	N4—C18—H18A	109.5
C3—C2—C1	125.1 (2)	N4—C18—H18B	109.5
C2—C3—C4	118.3 (2)	H18A—C18—H18B	109.5
C2—C3—H3B	120.9	N4—C18—H18C	109.5
C4—C3—H3B	120.9	H18A—C18—H18C	109.5
C5—C4—C3	119.4 (2)	H18B—C18—H18C	109.5
C5—C4—H4A	120.3	H1—O1W—H2	105.5
C3—C4—H4A	120.3	H3—O2W—H4	106.0
C4—C5—C6	118.7 (2)	H3—O2W—H5	110.2
C4—C5—H5C	120.6	H4—O2W—H5	109.9
C6—C5—H5C	120.6	H6—O3W—H7	110.0
N1—C6—C5	121.0 (2)	H8—O4W—H9	108.1
			
N1—Co1—O1—C1	2.34 (17)	C2—N1—C6—C5	−1.7 (4)
N2—Co1—O1—C1	−178.09 (18)	Co1—N1—C6—C5	179.89 (18)
O8—Co1—O1—C1	104.61 (18)	C2—N1—C6—C7	177.0 (2)
O4—Co1—O1—C1	0.0 (3)	Co1—N1—C6—C7	−1.4 (3)
O5—Co1—O1—C1	−102.69 (18)	C4—C5—C6—N1	0.7 (4)
N1—Co1—O4—C7	9.68 (17)	C4—C5—C6—C7	−177.8 (2)
N2—Co1—O4—C7	−170.03 (17)	Co1—O4—C7—O3	164.3 (2)
O8—Co1—O4—C7	−95.15 (17)	Co1—O4—C7—C6	−13.2 (3)
O5—Co1—O4—C7	113.32 (17)	N1—C6—C7—O3	−167.8 (2)
O1—Co1—O4—C7	12.1 (3)	C5—C6—C7—O3	10.9 (4)
N1—Co1—O5—C8	−164.94 (17)	N1—C6—C7—O4	10.0 (3)
N2—Co1—O5—C8	6.69 (17)	C5—C6—C7—O4	−171.4 (2)
O8—Co1—O5—C8	19.4 (3)	Co1—O5—C8—O6	177.3 (2)
O4—Co1—O5—C8	117.01 (18)	Co1—O5—C8—C9	−4.7 (3)
O1—Co1—O5—C8	−90.32 (18)	C13—N2—C9—C10	1.5 (4)
N1—Co1—O8—C14	172.30 (18)	Co1—N2—C9—C10	−173.89 (18)
N2—Co1—O8—C14	0.63 (18)	C13—N2—C9—C8	−176.7 (2)
O4—Co1—O8—C14	−111.46 (19)	Co1—N2—C9—C8	7.9 (3)
O5—Co1—O8—C14	−12.0 (3)	O6—C8—C9—N2	176.6 (2)
O1—Co1—O8—C14	95.48 (19)	O5—C8—C9—N2	−1.6 (3)
O8—Co1—N1—C6	81.22 (18)	O6—C8—C9—C10	−1.5 (4)
O4—Co1—N1—C6	−3.96 (17)	O5—C8—C9—C10	−179.7 (2)
O5—Co1—N1—C6	−96.65 (18)	N2—C9—C10—C11	−1.1 (4)
O1—Co1—N1—C6	177.18 (19)	C8—C9—C10—C11	176.9 (2)
O8—Co1—N1—C2	−97.19 (19)	C9—C10—C11—C12	−0.4 (4)
O4—Co1—N1—C2	177.62 (19)	C10—C11—C12—C13	1.3 (4)
O5—Co1—N1—C2	84.94 (19)	C9—N2—C13—C12	−0.5 (4)
O1—Co1—N1—C2	−1.23 (17)	Co1—N2—C13—C12	174.95 (18)
O8—Co1—N2—C9	178.3 (2)	C9—N2—C13—C14	179.3 (2)
O4—Co1—N2—C9	−98.58 (19)	Co1—N2—C13—C14	−5.2 (3)
O5—Co1—N2—C9	−7.92 (18)	C11—C12—C13—N2	−0.9 (4)
O1—Co1—N2—C9	80.44 (19)	C11—C12—C13—C14	179.3 (2)
O8—Co1—N2—C13	2.82 (17)	Co1—O8—C14—O7	175.7 (2)
O4—Co1—N2—C13	85.92 (19)	Co1—O8—C14—C13	−3.5 (3)
O5—Co1—N2—C13	176.59 (19)	N2—C13—C14—O7	−173.6 (2)
O1—Co1—N2—C13	−95.06 (18)	C12—C13—C14—O7	6.2 (4)
Co1—O1—C1—O2	176.8 (2)	N2—C13—C14—O8	5.6 (3)
Co1—O1—C1—C2	−3.0 (3)	C12—C13—C14—O8	−174.6 (2)
C6—N1—C2—C3	1.2 (4)	C18—N4—C15—N5	−11.3 (4)
Co1—N1—C2—C3	179.52 (18)	C16—N4—C15—N5	−179.8 (2)
C6—N1—C2—C1	−178.2 (2)	C18—N4—C15—N3	168.9 (2)
Co1—N1—C2—C1	0.2 (3)	C16—N4—C15—N3	0.4 (3)
O2—C1—C2—N1	−177.8 (2)	C17—N3—C15—N5	178.5 (2)
O1—C1—C2—N1	2.0 (3)	C17—N3—C15—N4	−1.7 (3)
O2—C1—C2—C3	2.9 (4)	C15—N4—C16—C17	0.9 (3)
O1—C1—C2—C3	−177.4 (2)	C18—N4—C16—C17	−167.8 (2)
N1—C2—C3—C4	0.4 (4)	C15—N3—C17—O9	−179.0 (3)
C1—C2—C3—C4	179.6 (2)	C15—N3—C17—C16	2.2 (3)
C2—C3—C4—C5	−1.3 (4)	N4—C16—C17—O9	179.4 (3)
C3—C4—C5—C6	0.8 (4)	N4—C16—C17—N3	−1.8 (3)

### Hydrogen-bond geometry (Å, °)

**Table:**

*D*—H···*A*	*D*—H	H···*A*	*D*···*A*	*D*—H···*A*
N3—H3A···O1W^i^	0.88	1.86	2.716 (3)	164
N5—H5A···O5	0.88	2.15	2.882 (3)	141
N5—H5B···O3^ii^	0.88	1.96	2.764 (3)	152
O1W—H1···O4^iii^	0.85	1.95	2.782 (3)	166
O1W—H2···O3W^iv^	0.85	1.85	2.673 (3)	164
O2W—H3···O4W^v^	0.85	1.70	2.522 (3)	163
O2W—H4···O6^vi^	0.85	1.64	2.481 (3)	170
O2W—H5···O2	0.85	1.71	2.537 (3)	164
O3W—H6···O7^iii^	0.85	1.93	2.778 (3)	172
O3W—H7···O9^vii^	0.85	2.22	2.948 (3)	144
O4W—H8···O7^viii^	0.85	1.84	2.680 (3)	169
O4W—H9···O1	0.85	1.87	2.718 (3)	172
C3—H3B···O9^vii^	0.95	2.37	3.301 (3)	165
C4—H4A···O8^ix^	0.95	2.43	3.252 (3)	145
C18—H18C···O7^iii^	0.98	2.60	3.535 (4)	160

Symmetry codes: (i) −*x*, −*y*+1, −*z*+1;  (ii) −*x*, −*y*+2, −*z*+1; (iii) *x*, *y*−1, *z*; (iv) *x*−1, *y*, *z*; (v) −*x*+1, −*y*+1, −*z*; (vi) *x*+1, *y*, *z*; (vii) −*x*+1, −*y*+1, −*z*+1; (viii) −*x*+1, −*y*+2, −*z*; (ix) −*x*+1, −*y*+2, −*z*+1.
